# Differential privacy medical data publishing method based on attribute correlation

**DOI:** 10.1038/s41598-022-19544-3

**Published:** 2022-09-21

**Authors:** Siqi Zhang, Xiaohui Li

**Affiliations:** School of Electronics and Information Engineering, Liaoning University of Technology, Jinzhou, 121000 China

**Keywords:** Energy science and technology, Mathematics and computing

## Abstract

The advent of the era of big data promotes the further development of medicine, and data release is an important step in it. The existing medical data release methods mostly use the k-anonymity model as the basis for data protection. With the advancement of technology, anonymous models are progressively less resistant to consistency attacks and background knowledge attacks. In order to better protect the private information of patients, this paper makes two major contributions: (1) The method of calculating the correlation between attributes is used to ensure the validity of the data after the data is released; (2) On the basis of the previous step, combined with the difference privacy-preserving model and tree model, this paper proposes an attribute association-based differential privacy classification tree data publishing method (ACDP-Tree). In this paper, simulation experiments are carried out on real medical data sets. The experimental results show that the algorithm ensures the validity and availability of the data to a certain extent while ensuring that the patient's privacy is not leaked.

## Introduction

**Figure 1:**
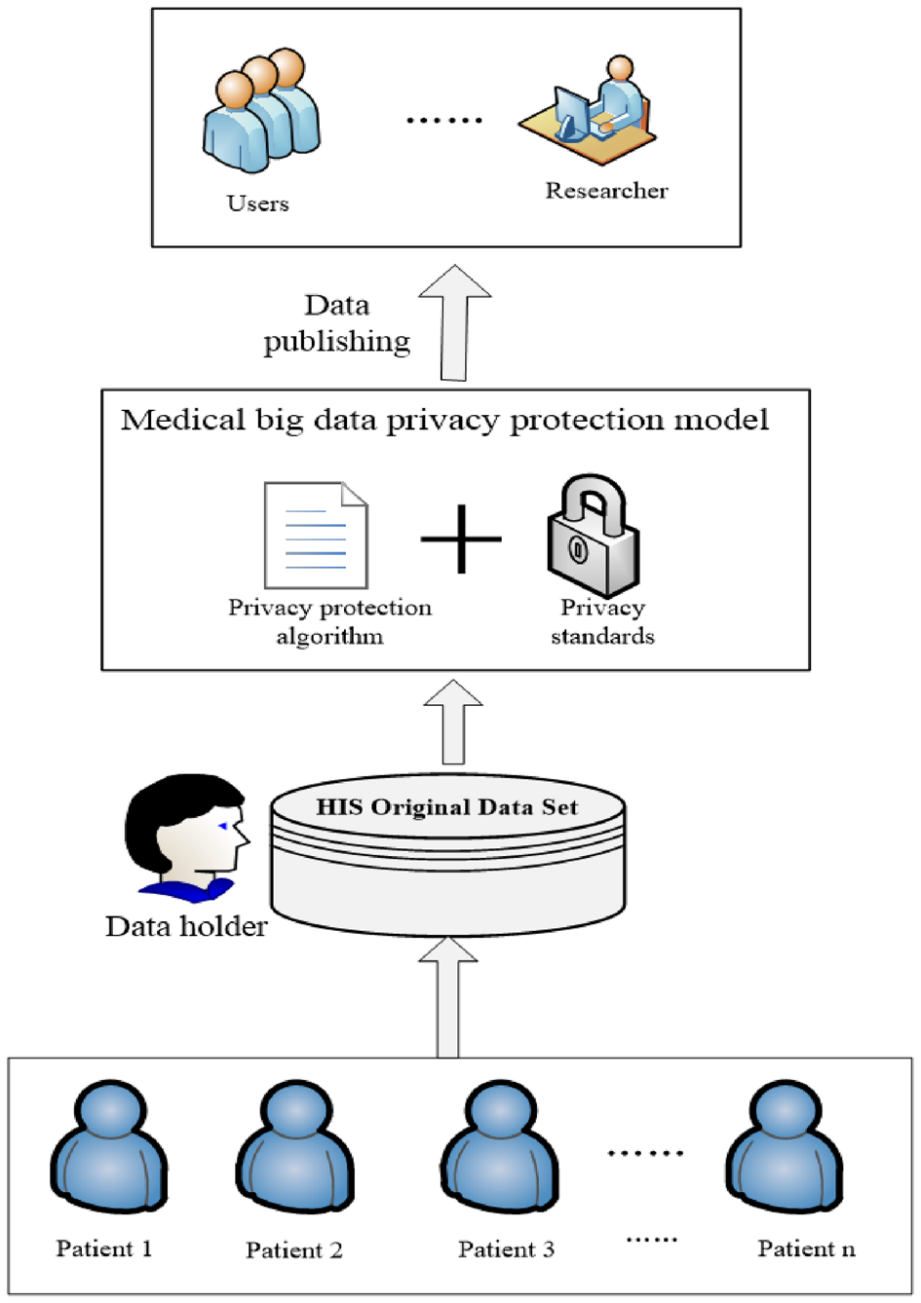
Medical big data privacy protection model.

The advent of the big data era has brought a lot of convenience to people's lives while also changing the way medical data is stored, and the traditional paper version of medical information is replaced by electronic medical record information; therefore, the Internet, cloud platforms, and blockchain^1^ are widely used to store various types of medical information. As shown in Fig. 1, patients inform their personal information to the doctor when they visit the hospital, and the doctor stores the patient's personal information into the hospital's database through consultation to form the original HIS dataset, which is managed and controlled by the hospital's database manager (data holder). Medical big data not only has the characteristics of big data (5 V)^2^, i.e., Volume, Velocity, Variability, Value, and Veracity. In addition to this, medical data is characterized by structural polymorphism, data information incompleteness, temporality, redundancy, and high sensitivity^3^.

With the progress of science and technology and the improvement of technology level, medical diagnosis means are gradually advanced, in order to better help patients to make diagnosis, various advanced medical equipment and diagnosis means are applied in the process of daily consultation, forming the characteristics of medical data modal diversity, including text-based information such as patients' personal consultation information, doctors' diagnosis results, patients' symptom descriptions, etc.; X-ray, CT and other kinds of imaging medical images, etc. There are text-based information such as patients' personal consultation information, doctors' diagnosis results and patients' symptom descriptions; picture-based information such as X-ray, CT and other types of medical images; signal spectrum-based information such as ECG and blood pressure graph; and video-based information such as ultrasound, ultrasound and surgery records. These data are constantly updated and changed with time and changes in patients' vital signs, so medical data have high timeliness and continuity, and the timeliness of medical data is reflected in the rescue of infected patients in the Corona Virus Disease. In addition, medical big data is also redundant. The same patient visits the same hospital for different diseases, and the system needs to re-enter the patient's personal information each time he or she registers, thus generating a large amount of redundant personal information, which will take a lot of time to pre-process the data, waste a lot of storage space, and reduce the availability of data. The incompleteness of medical big data is usually reflected in the process of data collection and processing. During the consultation process, the patient's description of his symptoms is unclear, the doctor's subjective judgment is incomplete and the diagnosis means are different, and other related factors will cause the incompleteness of data, and the consistency of data cannot be achieved.

Nowadays, the sharing of medical information and deeper medical research require data collectors to release the collected relevant medical information. Medical big data carries a large amount of citizens' personal information, which is highly sensitive compared with other big data, and medical consultation information mainly contains sensitive information such as ID card number, social security number, age, diseases suffered, drug dosage, attending doctors, etc. If the collected data is released directly, it will definitely cause the leakage of patients' private information and bring unnecessary troubles to patients. It is not enough to rely on national laws and policies to regulate medical institutions, but it is necessary to use scientific and technological means to protect the privacy of patients' medical information. To address this issue, researchers have proposed the concept of a privacy protection model for medical big data, which consists of privacy protection algorithms and privacy protection standards. The medical data processed by the model will be released, and the data will be analyzed and mined on the basis of satisfying the patient's privacy not to be disclosed. Therefore, protecting patients' privacy while ensuring data availability and security and releasing the data is a major research hotspot at present.

Privacy Preserving Data Distribution^4^ (PPDP) proposes different responses to different application scenarios. Traditional privacy-preserving models mostly use anonymous models such as k-anonymity^5^, l-diversity^6^, and t-closeness^7^ to anonymize data to achieve privacy protection. The basic idea of anonymous models is to achieve the effect of data indistinguishability and thus privacy protection by generalizing the data (i.e., replacing an exact value with a fuzzy range). For example, Leng et al.^8^ proposed the (w,k,d)-anonymity model, which takes advantage of the dual semantics of disease to build a semantic hierarchical tree and achieves the protection of sensitive attributes by restricting the average weight of equivalence classes and the average semantic distance; Jayapradha et al.^9^ proposed the QIAB-IMSB algorithm, which partitions the original dataset vertically to obtain QIAB (quasi-identifier bucket) and IMSB (individual multi-sensitive attribute bucket), and implement a hierarchical classification generalization method to anonymize these two buckets so that they satisfy k-anonymity and (k, l)-diversity, respectively; Gao et al.^10^ proposed the AOS algorithm, which selects a dimension to process the widest value domain by pre-defining the priority of the data mining task. The data is divided into two parts using median values and finally the respective anonymity completed data is merged and released; Khan et al.^11^ used absolute record similarity index to rank similar users, created and analyzed the equivalence classes by selecting appropriate parameters and finally generalized the data using global recoding approach.

However, the anonymous model cannot resist the attacker's consistency attack and background knowledge attack. Based on these problems, Dwork et al.^12^ proposed a differential privacy model, which does not depend on the attacker's knowledge of background, but is based on a rigorous mathematical basis to add noise to the query results or analysis results in order to achieve the effect of privacy protection. On top of that, Sun et al.^13^ proposed the DPDT algorithm, which combines differential privacy and decision trees to build a weight calculation system based on categorical regression trees, and in the process of building CART trees, the Gini index is calculated, the importance of attributes is analyzed, and the privacy budget is assigned according to the weights of the attributes; Lee et al.^14^ proposed the IPA algorithm, which combines differential privacy and anonymous method, which performs generalization, perturbation, and insertion operations on data, generates candidate datasets for data perturbation, and performs utility scoring on the candidate datasets, and selects suitable results for publication based on the final utility scoring results; Huirui Cao^15^ proposed a differential privacy data publication algorithm based on random forest, which uses random forest and standard matrix to calculate the strength of association between attributes, and based on the the correct rate of random forest identification and the correlation between attributes to assign privacy budgets, and publish the final results as histograms or statistical tables; Lin et al.^16^ proposed the MSDP (k, θ*, ε)-bounding model and proposed the MSDP_num_ anonymization algorithm based on this model, which uses the MS-bounding algorithm to aggregate data to construct QID group and then invoke the function for activation, and then add localized differential privacy noise satisfying ε within the QID group; Zheng et al^17^ proposed a privacy-preserving framework for data publishing, which invokes a data coordinator to fuse data and select data for publishing, a data owner to add Laplace noise to the data to be published for perturbation and sharing, and finally a data requester to generate data based on the sequence; Zhang et al^18^ proposed an LPPGE framework that combines differential privacy and adversarial learning together to achieve the ideal tradeoff between data availability and privacy preservation to some extent.

Although the above method satisfies the differential privacy with privacy budget of ε in the data publishing process, it does not take into account the association relationship between attributes and cannot provide value for subsequent data mining.

Based on the above analysis results, this paper proposes a differential privacy classification tree data publishing method (ACDP-Tree) based on attribute association. The contributions of this paper are as follows: first, the traditional hierarchical analysis method is improved to make it more applicable to medical data, and the improved method is used to evaluate the correlation of attributes among medical data and determine the attributes for iterative segmentation based on the strength of attribute correlation; second, based on the characteristics of the tree structure, the class equivocation method is used to assign the privacy budget so that it satisfies the sequence property and combination property of differential privacy.

## Basic theory and methods

### Table properties

#### **Definition 1**

(*Properties of a table*) Assuming a table dataset T, which contains t records (t is 9 in Table 1 below), each record corresponds to an entity, we mainly divide the attributes in the entity into three categories:

**Table 1:** Hospital medical record.

ID	Name	Age	Zipcode	Job	Disease
1	Alice	23	21,081	Doctor	Flu
2	Marry	26	22,081	Lawyer	Hepatitis
3	John	46	21,084	Teacher	HIV
4	Mike	42	21,015	Officer	Flu
5	Bob	32	22,074	Clerk	HIV
6	Jenny	37	21,071	Plumber	Hepatitis
7	Turman	55	21,009	Repairer	Pneumonia
8	Loary	48	22,003	Officer	Flu
9	Kitty	22	21,424	Teacher	Pneumonia

1. Identifier attribute^19^: can uniquely identify an individual (such as ID number);
2. Quasi-identification attribute: it cannot be used alone as an attribute field for identifying personal identity, and needs to be combined with other fields to achieve the purpose of unique identification (such as {Age}, {Zipcode}, {Job}, gender, zip code, etc. in Table 1) ;
3. Sensitive attributes: store personal sensitive information fields, which are the fields that attackers want to obtain (such as Table 1{Disease}, income, etc.).

### Differential privacy related concepts

Dwork et al.^12^ proposed a differential privacy protection model in 2006, which has a strict definition of privacy and is resistant to attacks from any background knowledge. The differential privacy protection model is based on data distortion technology. The basic principle of the model is to add appropriate noise values to the precise query or analysis results to perturb the results, so as to achieve the effect of protecting private data. This model ensures that the change of any record in the dataset will not affect the output result of the query. Therefore, the differential privacy protection model is widely used by researchers in various scenarios to protect the data. The following is a detailed description of differential privacy:

#### **Definition 2**

(*ε-differential privacy*^20^) For two data sets D1 and D2 that are identical or differ by one record, A is a random algorithm, and the output range of algorithm A to the data set Di is represented by Range (A), S Represents a subset of Range(A). If the algorithm satisfies the following formula (1), algorithm A is said to satisfy the differential privacy algorithm with a privacy budget of ε:

1 $${P}_{r}\left(A\left({D}_{1}\right)\in S\right)\le {e}^{\varepsilon }\times {P}_{r}\left(A\left({D}_{2}\right)\in S\right)$$

Among them, Pr(•) represents the output probability of algorithm A, ε is the privacy budget, which is used to measure the privacy protection degree of algorithm A. The magnitude of ε is inversely proportional to the degree of privacy protection, and the degree of privacy protection of algorithm A gradually becomes smaller as the value of ε increases.

#### **Definition 3**

*(global sensitivity*^21^*)*: Sensitivity is usually used to measure the impact of randomly deleting a record in a dataset on query results, denoted as *∆f*. Let the function have the following mapping relationship: *f:D → R*^*d*^, the input of the function is a data set, and the d-dimensional real number vector is used as the output result. For any adjacent datasets D_1_ and D_2_, the global sensitivity is expressed as the following Eq. (2),

2 $${GS}_{f}={\Vert {max}_{{D}_{1},{D}_{2}}f\left({D}_{1}\right)-f\left({D}_{2}\right)\Vert }_{1}$$

${\Vert {max}_{{D}_{1},{D}_{2}}f\left({D}_{1}\right)-f\left({D}_{2}\right)\Vert }_{1}$ where is denoted as 1-paradigm.

#### **Definition 4**

(*Privacy protection mechanism*^22^) Laplace mechanism and exponential mechanism are the two most representative privacy protection implementation mechanisms of the differential privacy protection model.

Adding random noise obeying the Laplace distribution to the precise query results is the basic principle of the Laplace mechanism, so as to achieve differential privacy protection that satisfies the privacy budget of *ε*. If the position parameter *Lap(b)* of the Laplace distribution is 0, then the probability density function expressed as:

3 $$\mathrm{p}\left(x\right)=\frac{1}{2b}{e}^{\left(-\frac{\left|x\right|}{b}\right)}$$

#### **Theorem 1**

(Laplace mechanism^23^) *For a data set D, if the function f:D → R*^*d*^*, and the global sensitivity of the function f is represented by ∆f, there is a random algorithm M(D) = f(D) + Y Provide ε-privacy protection, where Y obeys the Laplace distribution with parameter ∆f/ε, denoted as Y ~ Lap(∆f/ε).*

In view of the fact that the Laplace mechanism is only applicable to numerical query results, and in many practical applications, only numerical query results are often not enough to meet the needs, so the exponential mechanism is proposed to make up for the shortcomings of the Laplace mechanism, which is suitable for non-numeric queries result. The mechanism evaluates the quality of the output value *r* through the availability function *q*.

#### **Theorem 2**

(Exponential Mechanism^24^) *Let the random algorithm M input data set as D, r as the output entity object in the range of the value range Range(A), q(D, r) is defined as the availability function, ∆q corresponds to is the sensitivity of the function q(D,r), if the algorithm M selects and outputs r from the Range with a probability proportional to *${e}^{\frac{\varepsilon q\left(D,r\right)}{2\Delta q}}$* , then the algorithm M provides ε-differential privacy protection.*

### Concepts related to privacy protection

#### **Definition 5**

**Figure 2:**
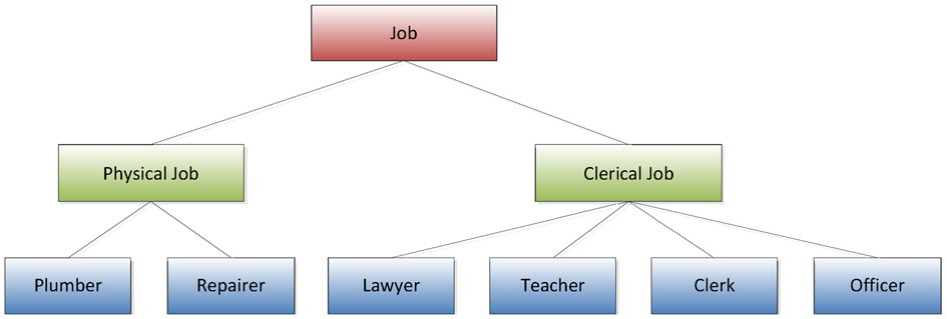
{Job} attribute generalization tree.

(*Attribute generalized tree*^25^) For the attribute domain *D* of a given attribute, where *D* is a finite set and *S* is used to represent the set of all nodes of a tree, then *S* = *{T,v1,v2….,vn ,s1,s2,….sn}*, (where *T* is the root node of the attribute generalized tree, *si* is the leaf node in the tree, and *vi* is other intermediate nodes that are not root and non-leaf nodes), let the function *f* be *S* to The mapping relationship of *D*, nodes a and b are two nodes in *S* that have a parent–child relationship, then there is *f(b) ⊆ f(a)*, and for the root node and leaf node: *f|si|*= *1 (1* ≤ *i* ≤ *n), f(s1) ∪ f(s2) ∪ ⋯ ∪ f(sn)* = *f(T) and f(T) ⊂ D*, for example, Fig. 2 is an attribute generalization tree of {Job} in Table 1.

#### **Definition 6**

(*Attribute correlation evaluation, ACE*) Analytic Hierarchy Process (AHP) decomposes complex target problems to obtain multiple targets or criteria, and then decomposes them into corresponding multiple levels, and calculates the hierarchical level through the fuzzy quantitative method of qualitative indicators, number and total order, and make objective multi-scheme optimization decisions. The traditional AHP method uses nine-level evaluation to evaluate attributes. For medical big data, the traditional AHP will increase the amount of calculation and increase the complexity and tediousness of the calculation. According to the characteristics of medical big data, the traditional analytic hierarchy process is improved, and the nine-level evaluation is simplified to three-level evaluation, which reduces the calculation amount to a certain extent and simplifies the calculation steps. The calculation of this method is divided into the following steps:

1. The first step is to establish a hierarchical structure model. According to the interaction among decision goals, decision criteria and decision objects, they are corresponding to the highest level, middle level and the lowest level in turn, and their hierarchical structure diagram is drawn;
2. *Constructing a judgment matrix:* compare the attributes in the attribute set *D* in turn, and the level needs to be evaluated according to the height of the generalization tree of the attribute hierarchy. *a*_*ij*_ is the result of the importance comparison between attribute i and attribute j. Attributes are composed of the result of quantification according to the height of the generalization tree of the attribute level, and the height of the smaller of the generalization tree heights of the two attribute levels is recorded as *h*. If *h* < *3*, it is recorded as slightly important, and the quantification value is 1. If *h* ≥ *6*, it is recorded as strongly important and the quantified value is 3; if *h* is in the middle of the two, it is recorded as strongly important and the quantified value is 2, as shown in Table 2. ${a}_{ij}=\frac{1}{{a}_{ji}}$ is one of the properties of the judgment matrix.

**Table 2:** Importance rating.

Attribute i and Attribute j	Quantized Value
Slightly important	1
Strong and important	2
Strongly important	3

(3) *Hierarchical single ordering and its consistency test: λ*_*max*_ is the largest eigenroot of the judgment matrix, *W* is the result vector of the normalization of the eigenvector corresponding to *λ*_*max*_, the ordering weight of the relative importance of a factor of the same level to a factor of the previous level Values are represented by elements in *W*, a process called hierarchical single ordering; the consistency check is defined as:
  4 $$\mathrm{CI}=\frac{\lambda -n}{n-1}$$

In the formula, *λ* is the largest characteristic root, and n is the order of the judgment matrix. When *CI* = *0*, the consistency effect is the best; the closer the *CI* is to 0, the better the consistency effect. The random consistency metric *RI* is used to measure the size of the *CI*:

5 $$\mathrm{RI}=\frac{{CI}_{1}+{CI}_{2}+\dots +{CI}_{n}}{n}$$

The above formula shows that the order *n* of the judgment matrix affects the value of the random consistency index *RI*. In general, with the increase of the order of the matrix, the probability of random deviation of consistency is also greater. For this reason, a test coefficient is introduced. The *CR* concept is used to test whether deviations in consistency are caused by random causes.

6 $$\mathrm{CR}=\frac{CI}{RI}$$

- (4) *Overall ranking of levels and consistency check:*calculate the weights of all factors that are relatively important to the highest level for all levels in turn, and perform consistency check.

### Methods and pseudo-code

In order to ensure the security of patients' personal information and the availability of subsequent data, this paper proposes a classification tree differential privacy data publishing algorithm (ACDP-Tree) based on attribute correlation. The algorithm first preprocesses the HIS original data set, deletes the identification attributes in the data set, generalizes each discrete quasi-identification attribute, builds an attribute generalization tree, and records the tree height *hi*; secondly, it uses the attribute correlation evaluation method Obtain the strength of the correlation between the quasi-identification attribute and the sensitive attribute (that is, the importance of the quasi-identification attribute to the sensitive attribute). The smaller the value, the higher the degree of privacy protection; for discrete data in the dataset, iterative segmentation is performed according to the attribute generalization tree, and the iterative segmentation ends when the attributes are divided to the next level of the generalization tree; for continuous data, the index mechanism is used to find the highest value. The optimal splitting point divides the value range into two, and iteratively divides it one by one. When the length of the split interval < *m* (a certain fixed value), the split ends.

For the allocation of the privacy budget, this algorithm first divides the privacy budget* ε* into two equal parts, and part of the privacy budget uses the class arithmetic method to reasonably allocate the privacy budget to each layer of the publication tree, and builds a tree model that conforms to the differential privacy feature. Another part of the privacy budget is used for the Laplacian mechanism, adding Laplacian noise to the leaf node count after the segmentation is completed, and publishing the leaf nodes after adding the noise.

The algorithm pseudo code is as follows.
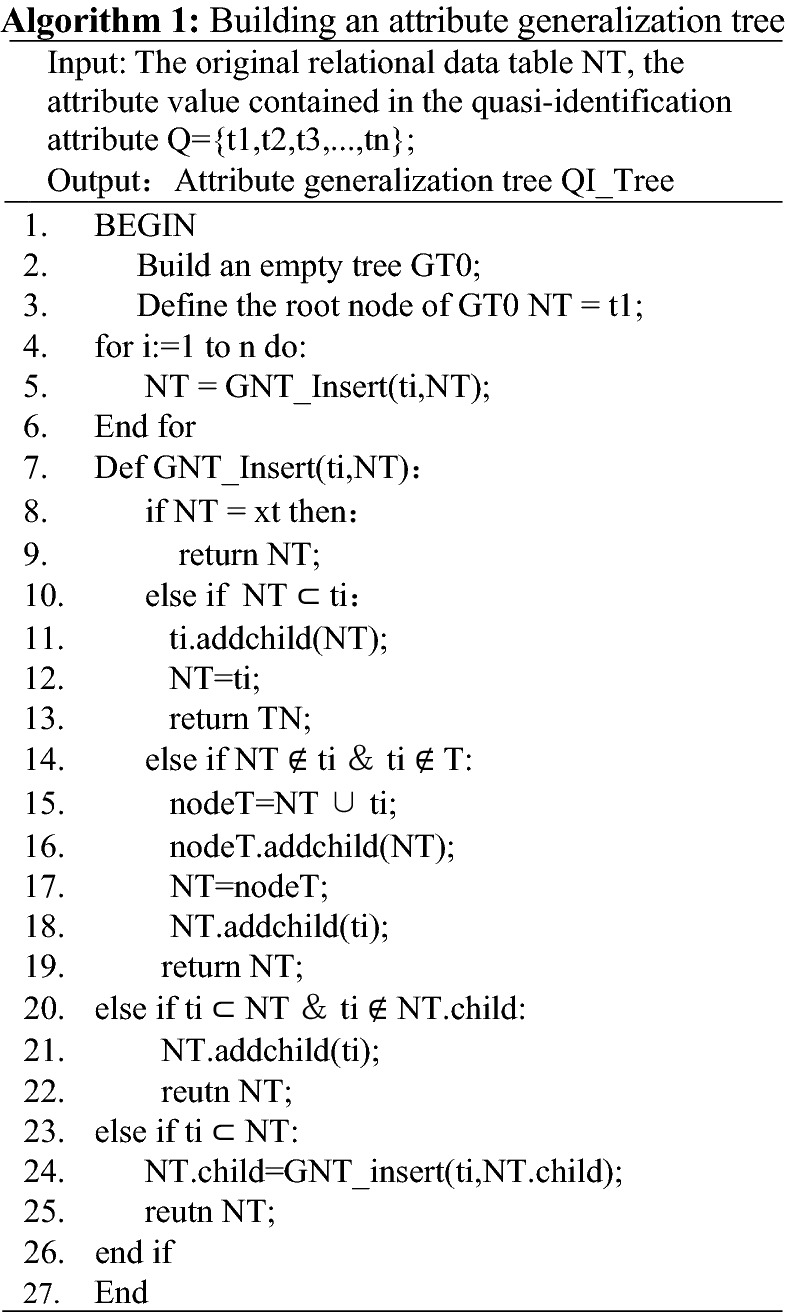


An explanation is given for the above algorithm. In Algorithm 1, the attribute set Q = {t1,t2,t3,…… ,tn} to construct a probabilistic tree of attributes, firstly, a tree structure is constructed, and a value of the attribute set Q is taken as the root node of the tree; then the next element attribute value of the attribute set is inserted in turn to form the parent node of the new tree, and all the elements of the attribute set are traversed, that is, the insertion is successful, and a probabilistic tree structure is constructed; finally, the function GNT_Insert(ti,NT) is called. The function mainly implements the function of constructing new nodes based on the probabilistic tree structure. The construction of new nodes is divided into the following cases: a). If the data to be inserted is equal to the data of the root node, no insertion operation is performed; b). When the data to be inserted contains the data of the root node, the data to be inserted is used as the new root node; c). If both the data to be inserted and the root node do not contain each other, the data to be inserted and the old root node are treated as children of the new root node; d). If the data of the root node contains the data of the node to be inserted and at the same time is not in the data of any of its children, the data to be inserted is inserted into the position of the new child node of the root node to be inserted; e). When the root node's child nodes contain the data to be inserted, then the recursive approach is used to insert the data to be inserted into one of the positions of the root node's sub-tree, and the other inserted nodes way, in accordance with this recursive approach in turn, until all the data are inserted.
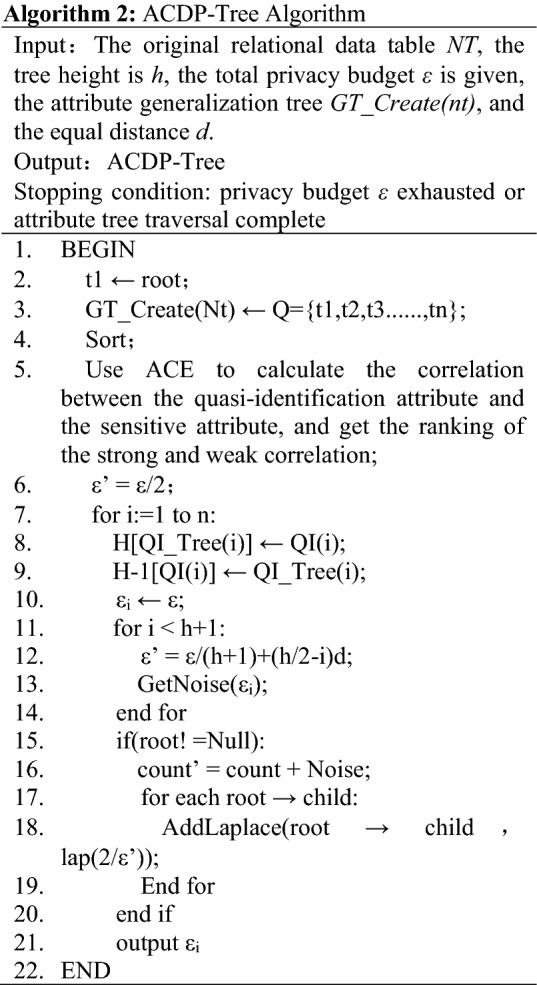


Algorithm 2 is an ACDP-Tree constructed on the basis of Algorithm 1. First, the total budget is divided into two parts. At the same time, the correlation between the quasi-identified attribute and the sensitive attribute is calculated by ACE, and the correlation ranking table is obtained. The sorted result assigns the privacy budget to the attributes and is used to construct a classification tree that conforms to the differential privacy characteristics. By adjusting the value of the parameter *d*, the distribution method of the equal-difference budget can be changed. When *d* is 0, the privacy budget allocated to each layer of the classification tree is the same. If *0* < *d* < *[h(h* + *1)]*, the difference of the privacy budget allocated from the root node to the leaf node is *d*. Finally, after the classification tree is formed, the remaining 1/2 of the privacy budget will be allocated to the statistical results of the leaf nodes in turn, and Laplacian noise will be added to it.

### Ethics approval and protection of personal information

The methods in this paper are in accordance with the relevant guidelines and provisions, the method of this paper in accordance with the "information security technology personal information security standards" proposed on the collection of personal information, storage, use, sharing, transfer, public disclosure and other links of the principles and security requirements; And meet the health Insurance Portability and Accountability Act (HIPPA) protection requirements for sensitive patient health information.

All the experimental schemes of this paper have been approved by National Information Security Standardization Technical Committee and China Institute of Electronic Technology Standardization According to the requirements of the UCI Machine Learning Data repository for donated data sets, all data sets used in this paper have obtained the informed consent of the data owner.

## Results and analysis

The experiment uses the Adult dataset provided by UCI as the real medical dataset. The Adult dataset is derived from the US Census database, which contains 48,842 records, each with 14 attributes, including 6 continuous attributes and 8 discrete attributes. In the experiments of this paper, age, marital-status, occupation and sex are selected as quasi-identification attributes, and capital-gain is selected as a sensitive attribute. Table 3 describes each attribute type. The algorithm proposed in this paper is written in the Python programming language and developed and implemented using the PyCharm integrated environment. The hardware environment of the experiment is AMD Ryzen 5 4600H with Radeon Graphics 3.00 GHz processor, 16G memory; Windows operating system.

**Table 3:** Adult attributes and attribute types.

Attributes	Type
Age	Continuous
Marital-status	Discrete
Occupation	Discrete
Sex	Discrete
Capital-gain	Continuous

In this section, the ACDP-Tree algorithm proposed in this paper is compared with the DPDT algorithm of Sun et al. and the IPA algorithm of Lee et al. Experiments are conducted to compare both the information loss degree and the absolute error, and to analyze the degree of privacy protection of the algorithm in this paper in terms of the data leakage risk probability:

### Information loss degree

Information loss mainly occurs in the data pre-processing and data generalization stages. In this paper, we use GLoss(n*) to measure the degree of information loss from attribute n to attribute n* with the following equation.

7 $$GLoss\left({n}^{*}\right)=\frac{{X}_{ST-{n}^{*}}}{{X}_{ST}}$$

Among them, ${{\varvec{X}}}_{{\varvec{S}}{\varvec{T}}}$ is the total number of leaf nodes of the attribute generalization tree, n* is the root node of a subtree, and ${{\varvec{X}}}_{{\varvec{S}}{\varvec{T}}-{{\varvec{n}}}^{\boldsymbol{*}}}$ is the total number of leaf nodes of the subtree.

**Figure 3:**
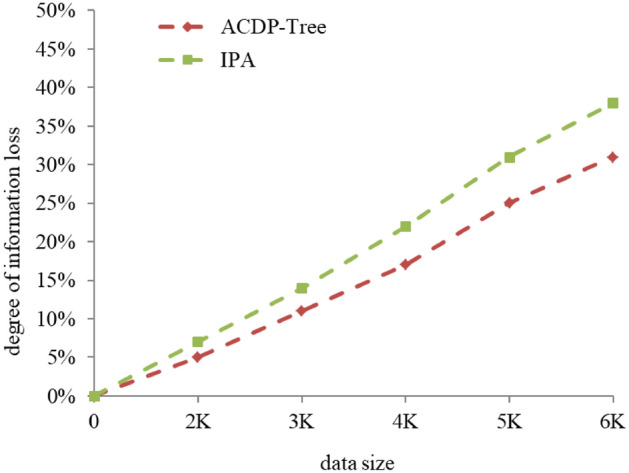
Changes in the degree of information loss with the size of the data.

Figure 3 gives a comparison between the ACDP-Tree algorithm of this paper and the IPA algorithm of Lee et al. in terms of information loss degree with the same privacy budget. As can be seen from Fig. 3, the information loss degree of both algorithms increases with the gradual increase of the data volume. However, under the condition of the same data volume, the privacy loss degree of this paper's algorithm is lower than that of IPA algorithm, mainly because when the data volume increases, the attribute values of the same attribute also gradually increase, and both of them use the method of full-domain generalization, and IPA algorithm combines the attribute classification trees to form a hierarchical lattice of attribute generalization, which leads to the higher information loss degree of IPA algorithm than this paper's algorithm. Therefore, overall, the algorithm in this paper is slightly better than the IPA algorithm.

### Absolute error

The absolute error^26^ is used to measure the absolute magnitude of the deviation of the published value after privacy protection from the true value of the query result. This paper measure the validity and availability of data by citing absolute error. The formula for calculating the absolute error is as follows:

8 $$\mathbf{E}\mathbf{r}\mathbf{r}\mathbf{o}\mathbf{r}=\left|{{\varvec{x}}}_{{\varvec{i}}}-{{\varvec{x}}}_{{\varvec{j}}}\right|$$

In the above formula, ${{\varvec{x}}}_{{\varvec{i}}}$ represents the real value of the query result, and ${{\varvec{x}}}_{{\varvec{j}}}$ represents the published value after privacy protection.

**Figure 4:**
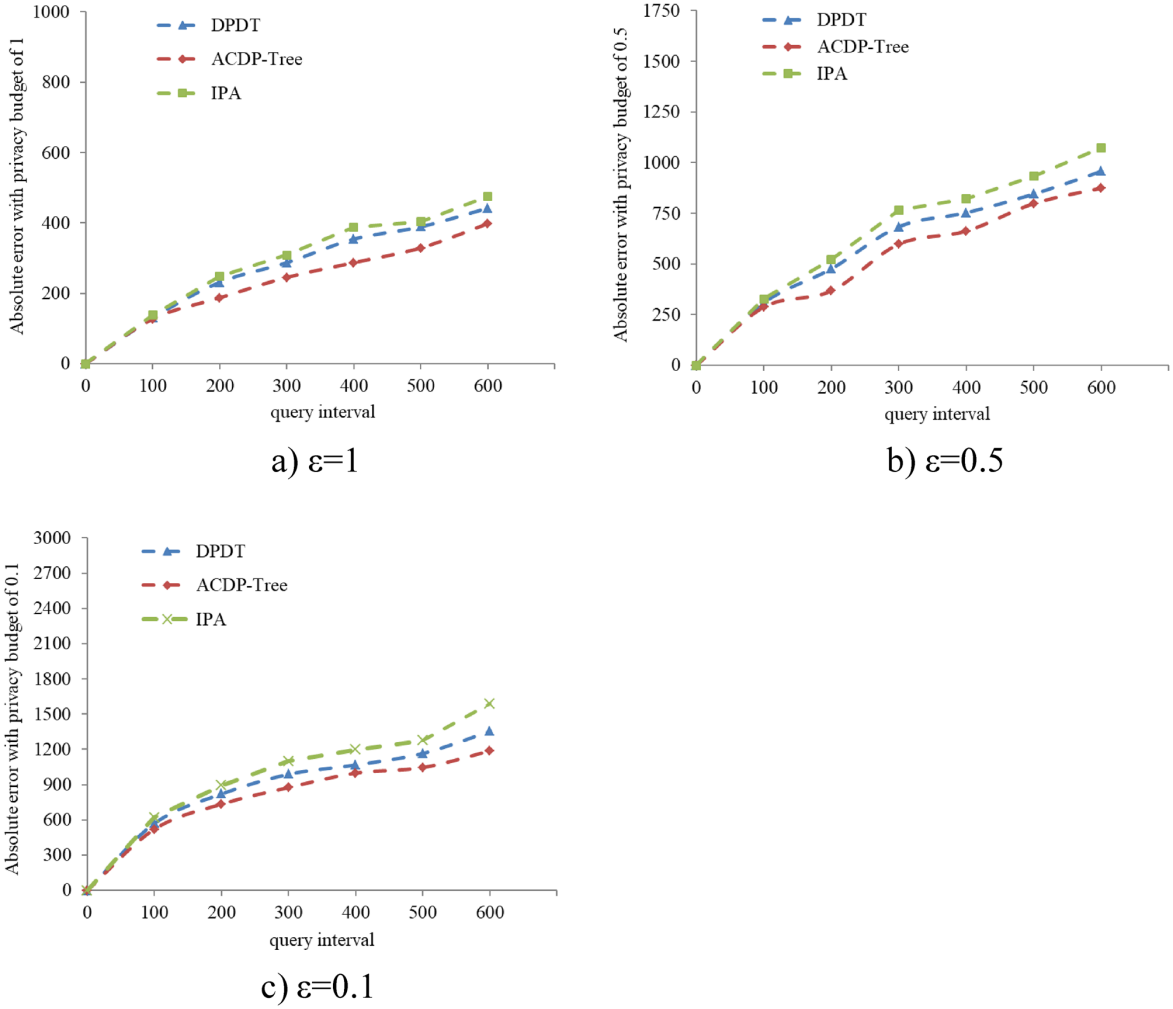
The change of absolute error when the query interval is 100 and ε takes different values.

The three graphs (a), (b), and (c) in Fig. 4 show the variations of the absolute errors of the ACDP-Tree algorithm proposed in this paper with the DPDT algorithm and IPA algorithm when ε is taken as 1,0.5,0.1, respectively, and we choose 100 as the interval of the query interval. As can be seen from the figure, when the privacy budget is the same, the absolute error increases gradually with the increase of the query interval; but when the query interval and the privacy budget are the same, the absolute error of ACDP-Tree is smaller than that of other The absolute error of the two algorithms. It can be concluded that the algorithm in this paper can effectively ensure the validity and availability of the data.

### Data leakage probability

Data leakage probability (DLP) is used to measure the degree of privacy protection of the algorithm; DLP reflects the number of records that are attacked during data distribution as a percentage of the total original data, and the smaller the value, the smaller the probability. Conversely, the higher the value, the higher the probability. The data leakage probability is calculated by the following formula.

9 $$\mathrm{DLP}=\frac{\left|{G}^{*}\right|}{\left|G\right|}$$

In the above equation,$\left|{G}^{*}\right|$ denotes the number of records under attack, $\left|G\right|$ denotes the number of records in the original data set, and 5 × 10^3^ records were randomly selected for this experiment.

**Figure 5:**
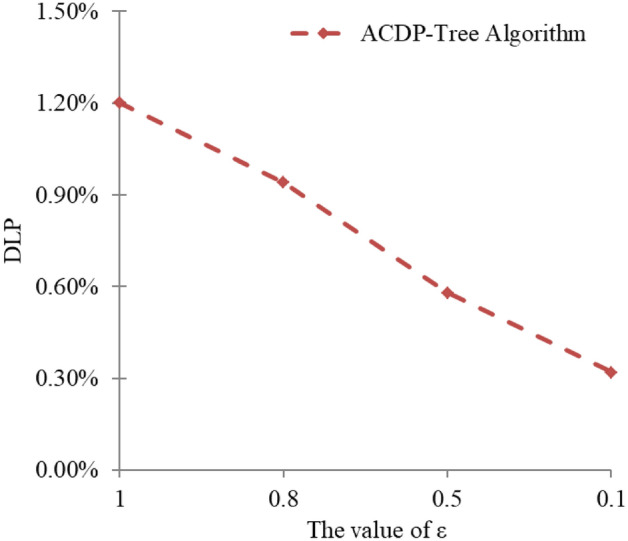
DLP variation trend under different privacy budgets.

Figure 5 shows the trend of data leakage probability when the privacy budget ε is taken as 1,0.5,0.1 respectively. From Fig. 5, it can be seen that as the value of ε decreases gradually, the DLP also decreases gradually. The main reason is that as the privacy budget value decreases, the degree of privacy protection of the differential privacy protection model gradually increases, which can better resist the background knowledge attack and the sensitive attribute similarity attack, making the attacker obtain less data during the attack, thus reducing the probability of data leakage and better protecting the user's privacy information.

### Time complexity analysis

The ACDP-Tree algorithm mentioned in this paper first needs to calculate the correlation strength of the quasi-identification attribute and the sensitive attribute according to the ACE, and sort the correlation strength. In the whole algorithm, the ACE only needs to be performed once, and the there is no need to perform calculation after the correlation strength is sorted. Therefore, the time complexity in this step is O(1); secondly, an attribute generalization tree needs to be created, assuming there are M quasi-identification attributes, each quasi-identification attribute There are n values, the time complexity required to build an attribute generalization tree is O(nlogn), corresponding to M quasi-identification attributes, the total time required is M•O(nlogn); The records are compressed to the root node, and the attributes are divided top-to-bottom in order from weak to strong according to the ranking of attribute correlation. If there are N data records, the time complexity of building a classification tree is O(NlogN).

### Space complexity analysis

Based on the algorithm proposed in this paper, a set of length M is firstly applied to store the quasi-identification attributes sorted according to the strength of attribute correlation, and the required space complexity is O(1); secondly, the tree structure does The required space complexity depends on the number of nodes in the tree, there are M quasi-identification attributes, each quasi-identification attribute has n values, and the space complexity required for the attribute generalization tree is M•O(n); Finally, for a classification tree for data distribution, with a total of N records, the required space complexity is O(N).

### Algorithm security analysis

In the process of data release, if there is a third-party malicious attack to obtain the data, firstly, the differential privacy model resists the attacker's powerful background knowledge attack with its strict mathematical definition, and the privacy of the record cannot be disclosed even if the attacker has all the records except one record; secondly, because ACDP-Tree iteratively partitions the attributes according to the ACE results and adds noise to the leaf node counts and then releases them, so the attacker cannot obtain the ACE results and thus cannot know the order of iterative attribute partitioning, so it is difficult for the attacker to infer the exact information of the users in the original dataset even if he obtains the data, thus achieving the effect of protecting patient privacy.

## Discussion

In the process of data release and sharing, medical data is prone to leak patient privacy and sensitive information, causing mental distress to patients and serious social panic. In response to the above problems, this paper proposes the ACDP-Tree differential privacy data publishing algorithm. ACDP-Tree perfectly combines the advantages of differential privacy and tree model, and adopts the idea of top-down iterative segmentation to construct an attribute summary for each quasi-identity attribute. On this basis, use ACE to select the best splitting attribute to iteratively split to construct a classification tree, and then use the class arithmetic method to allocate a corresponding privacy budget to each layer of the tree to satisfy the sequence composition and Parallel composition, and finally add Laplacian noise to leaf nodes and publish data. Through the comparative experiments, it can be seen that the ACDP-Tree differential privacy data publishing algorithm has the advantages of small absolute error, short program execution time consumption and little information loss. On the basis of protecting the patient's private information, the attribute correlation is preserved, and to a certain extent it provides convenience for subsequent data mining work.

## Data Availability

During the analysis of the current research data set in the UCI machine learning repository Adult data set in the http://archive.ics.uci.edu/ml/datasets/Adult online.
